# The SPI1/SMAD5 cascade in the promoting effect of icariin on osteogenic differentiation of MC3T3-E1 cells: a mechanism study

**DOI:** 10.1186/s13018-024-04933-3

**Published:** 2024-07-29

**Authors:** Junchao Zhang, Yi Mao, Jianwei Rao

**Affiliations:** 1 Department of Spine Surgery, The Quzhou Affiliated Hospital of Wenzhou Medical University, Quzhou People’s Hospital, No.100 Minjiang Avenue, Quzhou, 324000 Zhejiang P.R. China; 2 Department of Spine Surgery, Jiangshan People’s Hospital, Jiangshan, Quzhou, 324100 Zhejiang P.R. China

**Keywords:** Osteogenic differentiation, Icariin, MC3T3-E1 cells, SPI1, Autophagy

## Abstract

**Background:**

Dysregulation of osteogenic differentiation is a crucial event during osteoporosis. The bioactive phytochemical icariin has become an anti-osteoporosis candidate. Here, we elucidated the mechanisms underlying the promoting function of icariin in osteogenic differentiation.

**Methods:**

Murine pre-osteoblast MC3T3-E1 cells were stimulated with dexamethasone (DEX) to induce osteogenic differentiation, which was evaluated by an Alizarin Red staining assay and ALP activity measurement. The mRNA amounts of SPI1 and SMAD5 were detected by real-time quantitative PCR. Expression analysis of proteins, including osteogenic markers (OPN, OCN and RUNX2) and autophagy-associated proteins (LC3, Beclin-1, and ATG5), was performed by immunoblotting. The binding of SPI1 and the SMAD5 promoter was predicted by the Jaspar^2024^ algorithm and confirmed by chromatin immunoprecipitation (ChIP) experiments. The regulation of SPI1 in SMAD5 was examined by luciferase assays.

**Results:**

During osteogenic differentiation of MC3T3-E1 cells, SPI1 and SMAD5 were upregulated. Functionally, SPI1 overexpression enhanced autophagy and osteogenic differentiation of MC3T3-E1 cells, while SMAD5 downregulation exhibited opposite effects. Mechanistically, SPI1 could enhance SMAD5 transcription and expression. Downregulation of SMAD5 also reversed SPI1 overexpression-induced autophagy and osteogenic differentiation in MC3T3-E1 cells. In MC3T3-E1 cells under DEX stimulation, icariin increased SMAD5 expression by upregulating SPI1. Furthermore, icariin could attenuate SPI1 depletion-imposed inhibition of autophagy and osteogenic differentiation of MC3T3-E1 cells.

**Conclusion:**

Our findings demonstrate that the SPI1/SMAD5 cascade, with the ability to enhance osteogenic differentiation, underlies the promoting effect of icariin on osteogenic differentiation of MC3T3-E1 cells.

**Supplementary Information:**

The online version contains supplementary material available at 10.1186/s13018-024-04933-3.

## Highlights

SPI1 and SMAD5 are upregulated during MC3T3-E1 cell osteogenic differentiation.

SPI1 enhances SMAD5 transcription.

Icariin increases SMAD5 expression by upregulating SPI1.

The SPI1/SMAD5 axis underlies the promoting effect of icariin on MC3T3-E1 cell osteogenic differentiation.

**Supplementary Information:**

The online version contains supplementary material available at 10.1186/s13018-024-04933-3.

## Graphical abstract

In dexamethasone-stimulated murine pre-osteoblast MC3T3-E1 cells, icariin upregulates SPI1 and thus enhances SMAD5 transcription, leading to enhanced autophagy and osteogenic differentiation of MC3T3-E1 cells.

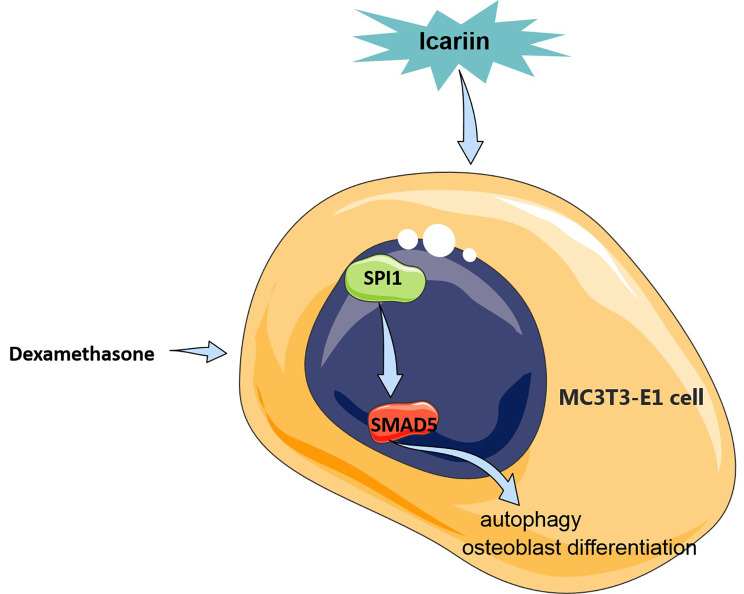

## Introduction

Osteoporosis, a frequent clinical condition characterized by reduced bone formation and decreased bone mass, poses a significant burden on morbidity and mortality in the global population aged over 60 years, leading to substantial healthcare costs [1]. In China, while the incidence is relatively lower compared with Caucasian populations, around 13% of individuals are affected by this disease [2]. Extensive efforts have been devoted to uncovering the pathophysiology of osteoporosis, with the imbalance of bone remodeling playing a key role in its development [3, 4]. Osteogenic differentiation is an essential process in bone regeneration, and its dysregulation is a crucial event in the pathogenesis of osteoporosis [5, 6]. The development of pharmacological agents based on enhancing osteogenic differentiation may provide novel opportunities for osteoporosis treatment [7].

The transcription factor SPI1, which recognizes specific binding sites within gene promoter, plays a significant role in regulating gene transcription, thereby functioning as critical regulators in various human diseases, such as Alzheimer’s disease and hepatocellular carcinoma [8, 9]. In the context of osteoporosis, bioinformatics analysis suggests that SPI1 could serve as a potential diagnostic marker [10]. Recent studies have demonstrated that SPI1 is capable of controlling osteogenic differentiation of bone marrow mesenchymal stem cells in acute suppurative osteomyelitis and ankylosing spondylitis fibroblasts [11, 12]. Nonetheless, the exact role of SPI1 in osteogenic differentiation largely remains to be elucidated.

SMAD5, a member of the receptor-activated SMADs that work as transcriptional effectors for the TGF-β signaling, has been implicated in a variety of physiopathological processes, including autophagy [13] and osteogenic differentiation [14, 15]. SMAD5 has been indicated to function as an osteoblast-related marker, and the promotion of SMAD5 phosphorylation contributes to osteoblast differentiation [16]. Moreover, recent evidence suggests that SMAD5 has close relevance to the pathogenesis of osteoporosis through its regulatory functions in osteogenic differentiation and bone formation [17, 18]. During bone remodeling in a rat model of diabetic osteoporosis, the upregulation of SMAD5 expression underlies the effect of the flavonoids of *Rhizoma Drynariae* (RDF) in promoting the formation of bone trabeculae [17]. Moreover, several miRNAs, such as miR-24-1-5p and miR-451a, regulate osteoblast function during osteoporosis by targeting SMAD5 [19, 20].

As a biologically active component of Epimedii herba, icariin has recently become an intriguing protective phytochemical due to its anti-oxidant, anti-inflammatory, anti-cancer, neuroprotective, and cardiovascular protective properties [21, 22]. Because of its low water solubility and weak membrane permeability, multiple strategies have also been developed to improve its bioavailability and efficacy by elevating its concentration in desired places [23]. Icariin has been reported to control bone metabolism [24], wherein it can induce osteogenic differentiation [25, 26]. Many studies have documented that icariin possesses protective functions in combating osteoporosis [27, 28]. As an example, icariin enhances the autophagy of senescent macrophages to relieve osteoporosis, depending on its anti-inflammatory function [29]. On the basis of the crucial enhancement on osteogenic differentiation and protective function in osteoporosis, more research is needed to unveil its molecular basis.

In this report, using dexamethasone (DEX)-stimulated murine pre-osteoblast MC3T3-E1 cells, we demonstrate that icariin facilitates osteogenic differentiation depending on its modulation in the SPI1/SMAD5 cascade. These data provide new evidence for the pharmacological application of icariin in treating osteoporosis.

## Materials and methods

### Cell culture and treatment

In this study, all assays were conducted with a murine pre-osteoblast MC3T3-E1 cell line (Subclone 14, Catalog No. CRL-2594, ATCC, Manassas, VA, USA), which was propagated under ATCC-provided conditions consisting of Minimum essential medium Alpha (α-MEM, Gibco, Uppsala, Sweden) enriched with 10% FBS (Biochrom KG, Berlin, Germany) at 37 °C at 5% carbon dioxide. To induce osteogenic differentiation, we stimulated MC3T3-E1 cells with 100 nM DEX (Selleck, Shanghai, China) supplemented with 52 µg/mL ascorbic acid (Macklin, Shanghai, China) and 10 mM β-glycerol phosphate (Selleck) as described [30]. For the concentration course experiment of icariin, we maintained MC3T3-E1 cells for 48 h in the media containing icariin (MCE, Shanghai, China) at a concentration of 0, 1, 10, 50, or 100 µM. To evaluate the efficacy of icariin, MC3T3-E1 cells were exposed to 50 µM of icariin for 48 h.

### Plasmids, shRNAs, and transfection

For SPI1 overexpression analysis, a SPI1 cDNA construct was generated by inserting a mouse SPI1 cDNA sequence into a pcDNA3.1(+) plasmid (VectorBuilder, Guangzhou, China) and subsequently transfected into MC3T3-E1 cells, in accordance with the producer’s recommendations (Thermo Fisher Scientific, Geel, Belgium), using Lipofectamine 3000. For depletion experiments, we obtained the mouse SPI1 specific shRNA (sh-SPI1), mouse SMAD5 specific shRNA (sh-SMAD5), and scrambled shRNA (sh-NC) from VectorBuilder and transfected the relevant shRNA into MC3T3-E1 cells under the use of Lipofectamine 3000 and protocols. Subsequent to transfection, MC3T3-E1 cells were processed for further treatment.

### Real-time quantitative PCR (qPCR)

MC3T3-E1 cells were exposed to DEX conditions (100 nM DEX supplemented with ascorbic acid and β-glycerol phosphate) for 0, 3, 7, and 14 d and subjected to the extraction of total RNA using the BeyoMag™ RNA Kit as suggested by the vendor (Beyotime, Shanghai, China). For cDNA generation, 500 ng of RNA was subjected to reverse transcription PCR with random hexamers and the PrimeScript Kit as described by the vendor (TaKaRa, Dalian, China). Real-time qPCR for mRNA analysis was carried out with the SYBR Premix ExTaq (TaKaRa) and specific primer sets for SPI1 (5’-TGGCAGGCCCTTCGATAAAA-3’-forward and 5’-GTTGGTCAGATCCCCTGCTT-3’-reverse), SMAD5 (5’-GAACCCTAAGCTCTGGGAACT-3’-forward and 5’-GTGCAAGTCCTCGACCATCC-3’-reverse), RUNX2 (5’-ACCAACCGAGTCATTTAAGGCT-3’-forward and 5’-AGGCTGTTTGACGCCATAGTC-3’-reverse), osteocalcin (OCN) (5’-GAACAGACAAGTCCCACACAGC-3’-forward and 5’-TCAGCAGAGTGAGCAGAAAGA-3’-reverse), osteopontin (OPN) (5’-AACCAGCCAAGGACTAACTACG-3’-forward and 5’-GCAGGCTGTAAAGCTTCTCCTCT-3’-reverse), or the endogenous control β-actin (5’-AGGGAAATCGTGCGTGACAT-3’-forward and 5’-TCCAGGGAGGAAGAGGATGC-3’-reverse).

### Osteoblastic differentiation assays

To study the impact on osteoblastic differentiation, MC3T3-E1 cells after the relevant transfection were maintained under DEX conditions for 7 days, which was accompanied by icariin treatment (50 µM, 48 h) or not. After that, we evaluated MC3T3-E1 cell osteoblastic differentiation by measuring calcium deposition, alkaline phosphatase (ALP) activity, and the amount of osteogenic markers (OPN, OCN, and RUNX2) by immunoblotting, as described below.

For the assessment of calcium deposition, we carried out the Alizarin Red staining assay as reported [31]. In brief, after fixation with 4% formaldehyde (Solarbio, Beijing, China), MC3T3-E1 cells were subjected to Alizarin Red (2%) staining (Solarbio), and followed by the application of cetylpyridinium chloride (Solarbio). We read absorbance at 570 nm under a Viktor X3 reader (Perkin Elmer, Turku, Finland) and obtained images using an Eclipse TS100 microscope (Nikon, Tokyo, Japan).

For the analysis of ALP activity, total extraction of MC3T3-E1 cells was processed by the ALP Assay Kit as recommended by the supplier (Beyotime), and the absorbance at 405 nm was proportional to the ALP activity. Meantime, we conducted ALP staining with the Beyotime ALP Color Development Kit following the concomitant instructions.

### Immunoblotting

Lysates of MC3T3-E1 cells after the indicated treatment or/and transfection were prepared with the lysis buffer (10 mM Tris, pH = 7.4, 150 mM NaCl, 0.1% SDS, 1% deoxycholate, 1% NP-40, and a protease inhibitor cocktail (Beyotime). After 10% SDS gel electrophoresis and nitrocellulose transfer (Servicebio, Wuhan, China), protein expression analyses were carried out as described [18], using specific antibodies. With the exception of rabbit polyclonal to OCN (Catalog No. PA5-96529, 1 to 800) from Thermo Fisher Scientific, we purchased rabbit monoclonal to SPI1 (Catalog No. ab227835, 1 to 1,000), rabbit monoclonal to SMAD5 (Catalog No. ab40771, 1 to 4,000), rabbit recombinant multiclonal to OPN (Catalog No. ab307994, 1 to 300), rabbit monoclonal to RUNX2 (Catalog No. ab236639, 1 to 1,000), rabbit polyclonal to LC3I/II (Catalog No. ab48394, 1 to 1,500), rabbit monoclonal to Beclin-1 (Catalog No. ab207612, 1 to 2,000), rabbit monoclonal to ATG5 (Catalog No. ab108327, 1 to 5,000), and rabbit polyclonal to β-actin (Catalog No. ab8227, 1 to 3,000) from Abcam (Cambridge, UK).

### Prediction of the binding of SPI1 and the SMAD5 promoter

Prediction analysis for the binding of SPI1 with the SMAD5 promoter was implemented by the Jaspar^2024^ algorithm at https://jaspar.elixir.no/.

### Chromatin immunoprecipitation (ChIP) experiments

The binding of SPI1 with the SMAD5 promoter was confirmed using the ChIP Assay Kit (Gene Create, Wuhan, China) and the anti-SPI1 antibody (Catalog No. ab227835, 1 µg for 5 µg of chromatin, Abcam). Briefly, after MC3T3-E1 cells were cross-linked with 1% formaldehyde, chromatin DNA preparation was conducted as per manufactory protocols, which was followed by chromatin fragmentation (200–700 bp). Through co-incubation with Protein A + G magnetic beads, chromatin fragments were precipitated under the use of the anti-SPI1 or isotype anti-IgG (Catalog No. ab172730, Abcam) antibody. DNA was obtained from the SPI1-associating precipitates and subjected to qPCR for the assessment of the amount of the SMAD5 promoter.

### Luciferase assays

To generate SMAD5 reporters, we cloned the SMAD5 promoter fragment encompassing the putative binding site (CAGTTGAGGAAGTGAAA) (SMAD5-WT) or mutated seed sequence CACAACTCCTTCACTAA (SMAD5-MUT) into the pGL3 basic vector (Miaoling, Wuhan, China). MC3T3-E1 cells were subjected to co-transfection of SMAD5-WT or SMAD5-MUT and pcDNA control or a SPI1 cDNA plasmid using Lipofectamine 3000. As the normalization control, the *Renilla* luciferase plasmid (Promega, Charbonnières, France) was also used. Cell extract preparation was performed at 48 h post-transfection, and luciferase activities (firefly and *Renilla*) were analyzed with the Dual-Luciferase System provided by Promega.

### Statistical analysis

Data from at least three replicates (*n* ≥ 3) were expressed as mean ± s.d. We analyzed statistical difference between groups (*P* < 0.05 was considered significant) using an unpaired Student’s *t*-test or ANOVA (one- or two-way) when appropriate.

## Results

### **Increased expression of SPI1 during osteogenic differentiation of MC3T3-E1 cells**

**Fig. 1:**
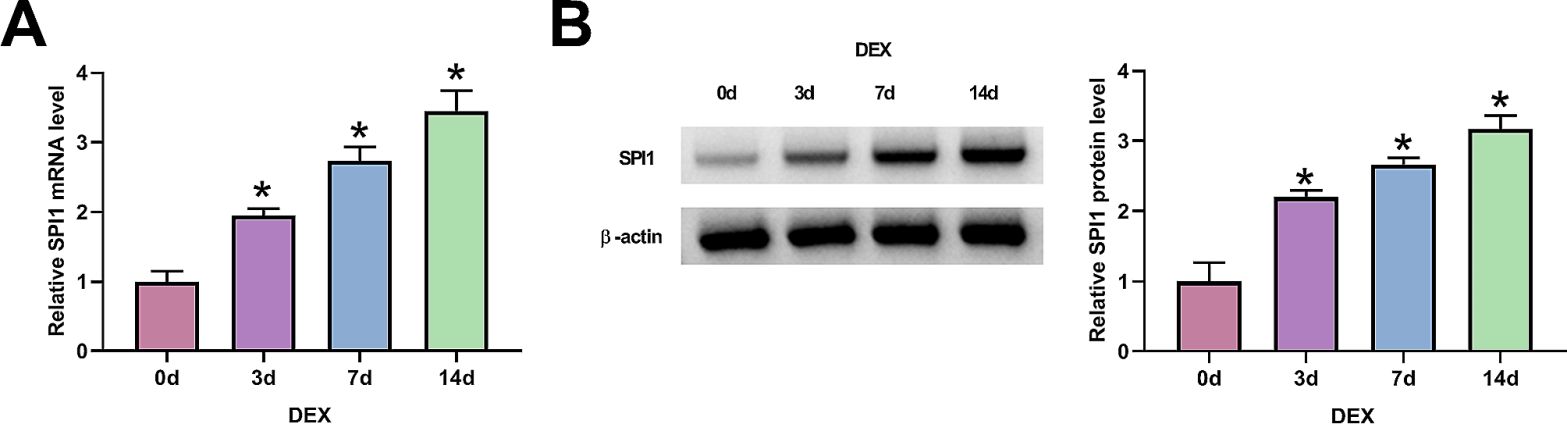
Upregulation of SPI1 during osteogenic differentiation of MC3T3-E1 cells. **(A-B)** The levels of SPI1 mRNA (real-time qPCR) **(A)** and protein (immunoblotting) **(B)** in MC3T3-E1 cells exposed to 100 nM DEX supplemented with ascorbic acid and β-glycerol for 0, 3, 7 and 14 d. *n* = 3 independent biological replicates in A and B. *P < 0.05

It has been demonstrated that the transcription factor SPI1 has key functions during osteogenic differentiation of bone marrow mesenchymal stem cells and ankylosing spondylitis fibroblasts [11, 12]. In order to examine whether SPI1 is involved in osteogenic differentiation of MC3T3-E1 cells, we exposed cells to 100 nM DEX supplemented with ascorbic acid and β-glycerol phosphate (hereinafter referred to as DEX), which has been proven to induce osteogenic differentiation of MC3T3-E1 cells [30]. During osteogenic differentiation on 3, 7, and 14 d, we observed the progressively and significantly increased expression of SPI1 in MC3T3-E1 cells, as evaluated by real-time quantitative PCR (qPCR) and immunoblotting (Fig. 1A and B). These data suggest a close association between SPI1 upregulation and osteogenic differentiation in MC3T3-E1 cells.

### Increased expression of SPI1 induces autophagy and osteogenic differentiation in MC3T3-E1 cells

**Fig. 2:**
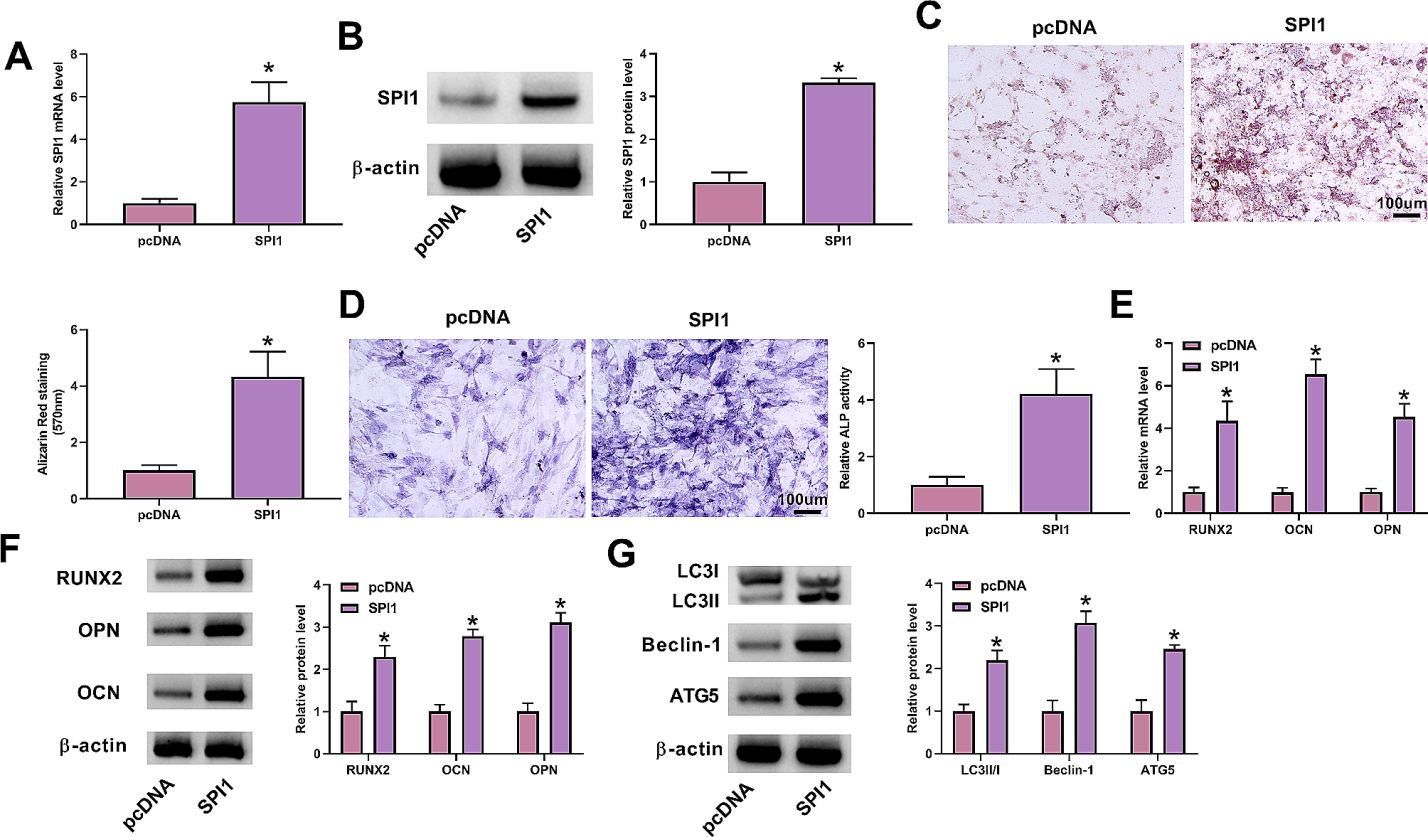
Functions of SPI1 in inducing autophagy and osteogenic differentiation of MC3T3-E1 cells. **(A and B)** The amount of SPI1 mRNA (real-time qPCR) and protein (immunoblotting) in MC3T3-E1 cells transfected with a SPI1 cDNA construct or pcDNA control before DEX stimulation. **(C-G)** The effects of SPI1 gain-of-function on in vitro calcium deposit (Alizarin Red staining assay) **(C)**, ALP activity (ALP activity assay) **(D)**, the expression of osteogenic markers (OPN, OCN and RUNX2) (real-time qPCR and immunoblotting) **(E and F)**, and the changes of LC3II/LC3I ratio and Beclin-1 and ATG5 levels (immunoblotting) **(G)**. *n* = 3 independent biological replicates in A-G. *P < 0.05

Accordingly, we elucidated the functions of SPI1 in osteogenic differentiation of MC3T3-E1 cells in vitro assays. To this purpose, a SPI1 cDNA construct was transfected into MC3T3-E1 cells before DEX stimulation. The upregulation efficiency of this construct was validated by qPCR and immunoblotting (Fig. 2A and B). Using the Alizarin Red staining assay, we observed a greater calcium deposit in SPI1-overexpressed MC3T3-E1 cells compared with the control cells (Fig. 2C). Similar results were observed with ALP activity, as evidenced by the enhanced ALP activity in SPI1-overexpressed MC3T3-E1 cells (Fig. 2D). We then monitored the expression of osteogenic markers (OPN, OCN, and RUNX2) in transfected MC3T3-E1 cells. In agreement with the above findings, MC3T3-E1 cells with increased SPI1 expression displayed higher levels of OPN, OCN, and RUNX2 than control cells (Fig. 2E and F). Induction of autophagy has been claimed to drive osteogenic differentiation of MC3T3-E1 cells [32, 33]. To support our findings, we also tested the changes in autophagy-associated proteins (LC3, Beclin-1, and ATG5) in transfected MC3T3-E1 cells. Via the immunoblotting assay, an enhanced ratio of LC3II/LC3I and an elevated expression of Beclin-1 and ATG5 were observed in SPI1-overexpressed MC3T3-E1 cells compared with pcDNA controls (Fig. 2G). These results together demonstrate that increased SPI1 expression is able to induce osteogenic differentiation in MC3T3-E1 cells.

### SPI1 enhances SMAD5 transcription in MC3T3-E1 cells

**Fig. 3:**
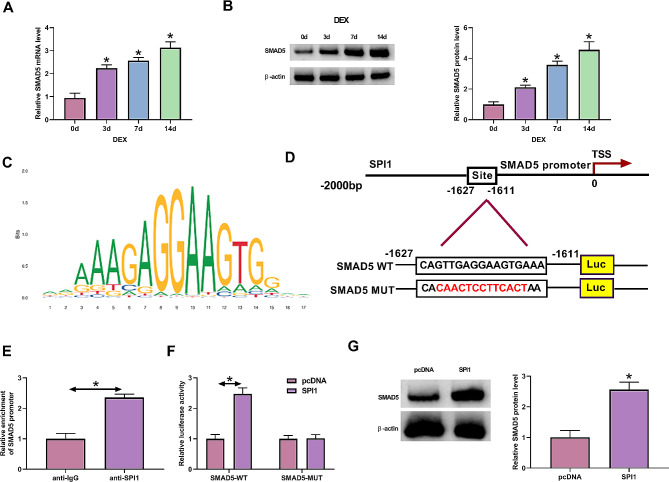
SPI1 enhances SMAD5 transcription and expression. **(A-B)** The levels of SMAD5 mRNA (real-time qPCR) **(A)** and protein (immunoblotting) **(B)** in MC3T3-E1 cells exposed to 100 nM DEX supplemented with ascorbic acid and β-glycerol for 0, 3, 7 and 14 d. **(C and D)** The motif of the SPI1 (C), the putative binding site (CAGTTGAGGAAGTGAAA) of SPI1 with the SMAD5 promoter (predicted by Jaspar^2024^ algorithm), and its mutation (CACAACTCCTTCACTAA) **(D)**. **(E)** ChIP experiments with lysates of MC3T3-E1 cells using anti-SPI1 or anti-IgG antibody confirmed the binding of SPI1 with the SMAD5 promoter. **(F)** Luciferase assays of MC3T3-E1 cells co-transfected with SMAD5-WT or SMAD5-MUT and pcDNA control or the SPI1 cDNA construct. **(G)** The amount of SMAD5 protein (immunoblotting) in MC3T3-E1 cells transfected with a SPI1 cDNA construct or pcDNA control. *n* = 3 independent biological replicates in A-G. *P < 0.05

Transcription factors control gene transcription and thus play significant roles in human disorders, including osteoporosis [34]. To uncover one mechanism by which SPI1 induces osteogenic differentiation in MC3T3-E1 cells, we considered its target genes that are tightly associated with osteoporosis pathogenesis and osteogenic differentiation. SMAD5 has been proven to facilitate osteogenic differentiation [14, 15] and participate in osteoporosis pathogenesis [17]. Consistent with SPI1 expression, increased levels of SMAD5 mRNA and protein were observed in MC3T3-E1 cells during osteogenic differentiation on 3, 7, and 14 d (Fig. 3A and B). In-depth bioinformatics analysis of the Jaspar^2024^ algorithm indicated the motif of SPI1 and predicted the possible binding of SPI1 and the SMAD5 promoter (Fig. 3C and D). The interaction of SPI1 with SMAD5 was confirmed by ChIP and luciferase assays. ChIP experiments with an anti-SPI1 antibody validated the binding of SPI1 with the SMAD5 promoter, as presented by the abundant enrichment of the SMAD5 promoter in the SPI1-associating complex (Fig. 3E). When the fragment of the SMAD5 promoter encompassing the putative binding site (CAGTTGAGGAAGTGAAA) was cloned into the pGL3 basic vector (SMAD5-WT), luciferase assays showed elevated luciferase activity following SPI1 overexpression (Fig. 3F), demonstrating that SPI1 can promote SMAD5 transcription. The promotion of SPI1 in SMAD5 transcription via the binding site was further demonstrated by the observation that luciferase activity was not altered by SPI1 overexpression when the binding site was mutated to CACAACTCCTTCACTAA (SMAD5-MUT) (Fig. 3F). In addition, we also examined the regulation of SPI1 in SMAD5 protein expression. In MC3T3-E1 cells, increased expression of SPI1 led to a striking elevation in SMAD5 protein expression (Fig. 3G). Collectively, these results provide strong evidence that SPI1 enhances SMAD5 transcription and expression.

### **Downregulation of SMAD5 diminishes autophagy and osteogenic differentiation in MC3T3-E1 cells**

**Fig. 4:**
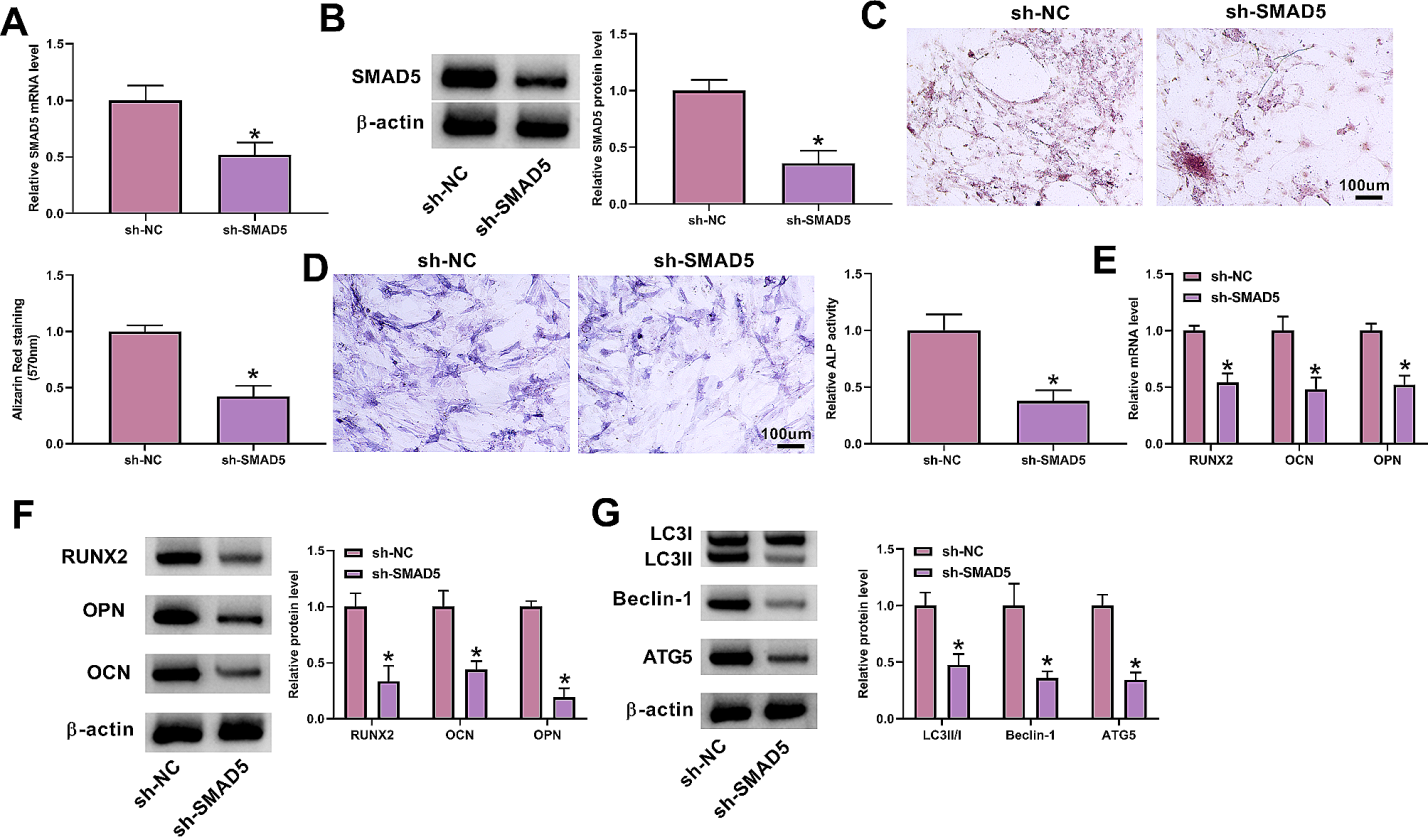
Impacts of SMAD5 downregulation on autophagy and osteogenic differentiation of MC3T3-E1 cells. **(A and B)** The evaluation of SMAD5 protein amount (real-time qPCR and immunoblotting) in MC3T3-E1 cells transfected with sh-NC or sh-SMAD5 before DEX stimulation. **(C-G)** The impacts of SMAD5 loss-of-function in the presence of DEX stimulation on in vitro calcium deposit (Alizarin Red staining assay) **(C)**, ALP activity (ALP activity assay) **(D)**, the levels of osteogenic markers (OPN, OCN and RUNX2) (real-time qPCR and immunoblotting) **(E and F)**, and the changes of LC3II/LC3I ratio and Beclin-1 and ATG5 levels (immunoblotting) **(G)**. *n* = 3 independent biological replicates in A-G. *P < 0.05

We next confirmed the functions of SMAD5 in osteogenic differentiation of MC3T3-E1 cells under DEX conditions. A SMAD5 specific shRNA (sh-SMAD5) was used to deplete SMAD5 in MC3T3-E1 cells in the presence of DEX stimulation (Fig. 4A and B). Remarkably, after SMAD5 downregulation, we observed reduced calcium deposit (Fig. 4C), shown by Alizarin Red staining, and suppressed ALP activity (Fig. 4D) in MC3T3-E1 cells under DEX conditions. Under DEX conditions, SMAD5-depleted MC3T3-E1 cells also displayed reduced levels of osteogenic markers OPN, OCN, and RUNX2 compared with control cells (Fig. 4E and F). In addition, immunoblotting assays further indicated the decrease of the LC3II/LC3I ratio and Beclin-1 and ATG5 levels in SMAD5-depleted MC3T3-E1 cells in the presence of DEX stimulation (Fig. 4G). All these data support, and consistent with earlier work [35, 36], the crucial role of SMAD5 in the regulation of osteogenic differentiation of MC3T3-E1 cells.

### Downregulation of SMAD5 reverses SPI1-induced autophagy and osteogenic differentiation in MC3T3-E1 cells

**Fig. 5:**
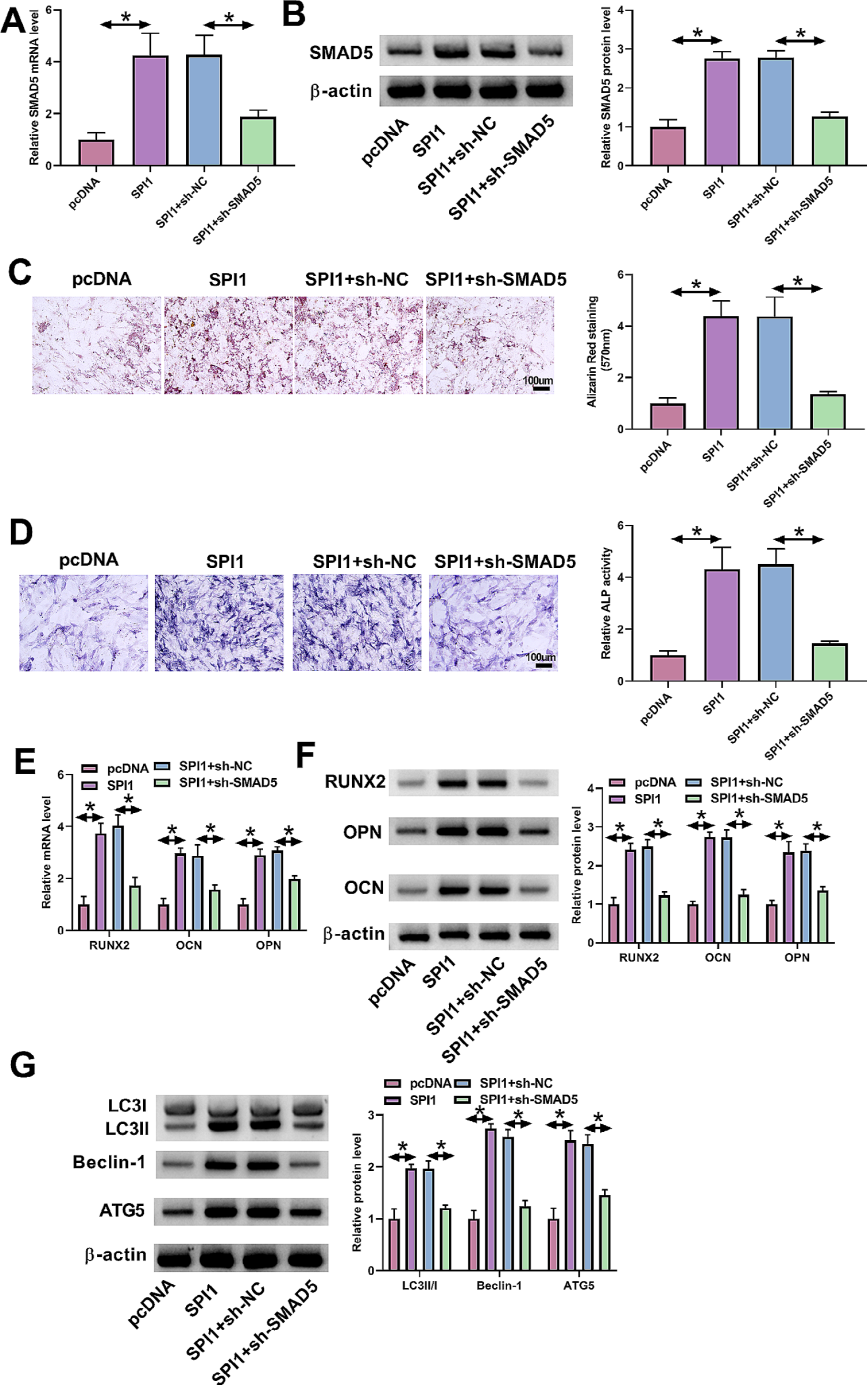
The SPI1/SMAD5 cascade induces autophagy and osteogenic differentiation of MC3T3-E1 cells. **(A and B)** The evaluation of SMAD5 mRNA (real-time qPCR) and protein (immunoblotting) amount in MC3T3-E1 cells after transfection of pcDNA, pcDNA-SPI1, pcDNA-SPI1 + sh-NC, or pcDNA-SPI1 + sh-SMAD5 before DEX stimulation. **(C-G)** MC3T3-E1 cells were transfected with pcDNA, pcDNA-SPI1, pcDNA-SPI1 + sh-NC, or pcDNA-SPI1 + sh-SMAD5 and checked for calcium deposit (Alizarin Red staining assay) **(C)**, ALP activity (ALP activity assay) **(D)**, the levels of osteogenic markers (OPN, OCN and RUNX2) **(E and F)**, and the changes of LC3II/LC3I ratio and Beclin-1 and ATG5 levels (immunoblotting) **(G)**. *n* = 3 independent biological replicates in A-G. *P < 0.05

The above observations suggest that SPI1 is able to enhance SMAD5 transcription in MC3T3-E1 cells. Accordingly, we further examined whether upregulated SPI1 induces osteogenic differentiation of MC3T3-E1 cells via the increase of SMAD5. We thus lessened SMAD5 expression using sh-SMAD5 in SPI1-overexpressed MC3T3-E1 cells, which exhibited high SMAD5 expression compared with control cells (Fig. 5A and B). Overexpression of SPI1 in MC3T3-E1 cells resulted in enhanced calcium deposit and ALP activity, which could be counteracted by lessened SMAD5 expression (Fig. 5C and D). Lessened expression of SMAD5 also abolished SPI1 overexpression-induced augmentation of osteogenic markers OPN, OCN, and RUNX2 in MC3T3-E1 cells (Fig. 5E and F). In addition, in MC3T3-E1 cells, SMAD5 downregulation exerted a counteracting impact on SPI1 overexpression-triggered enhanced changes of the LC3II/LC3I ratio and Beclin-1 and ATG5 levels (Fig. 5G). In conclusion, these findings demonstrate that SPI1 promotes osteogenic differentiation of MC3T3-E1 cells through the upregulation of SMAD5.

### Icariin attenuates SPI1 depletion-imposed inhibition of autophagy and osteogenic differentiation of MC3T3-E1 cells

**Fig. 6:**
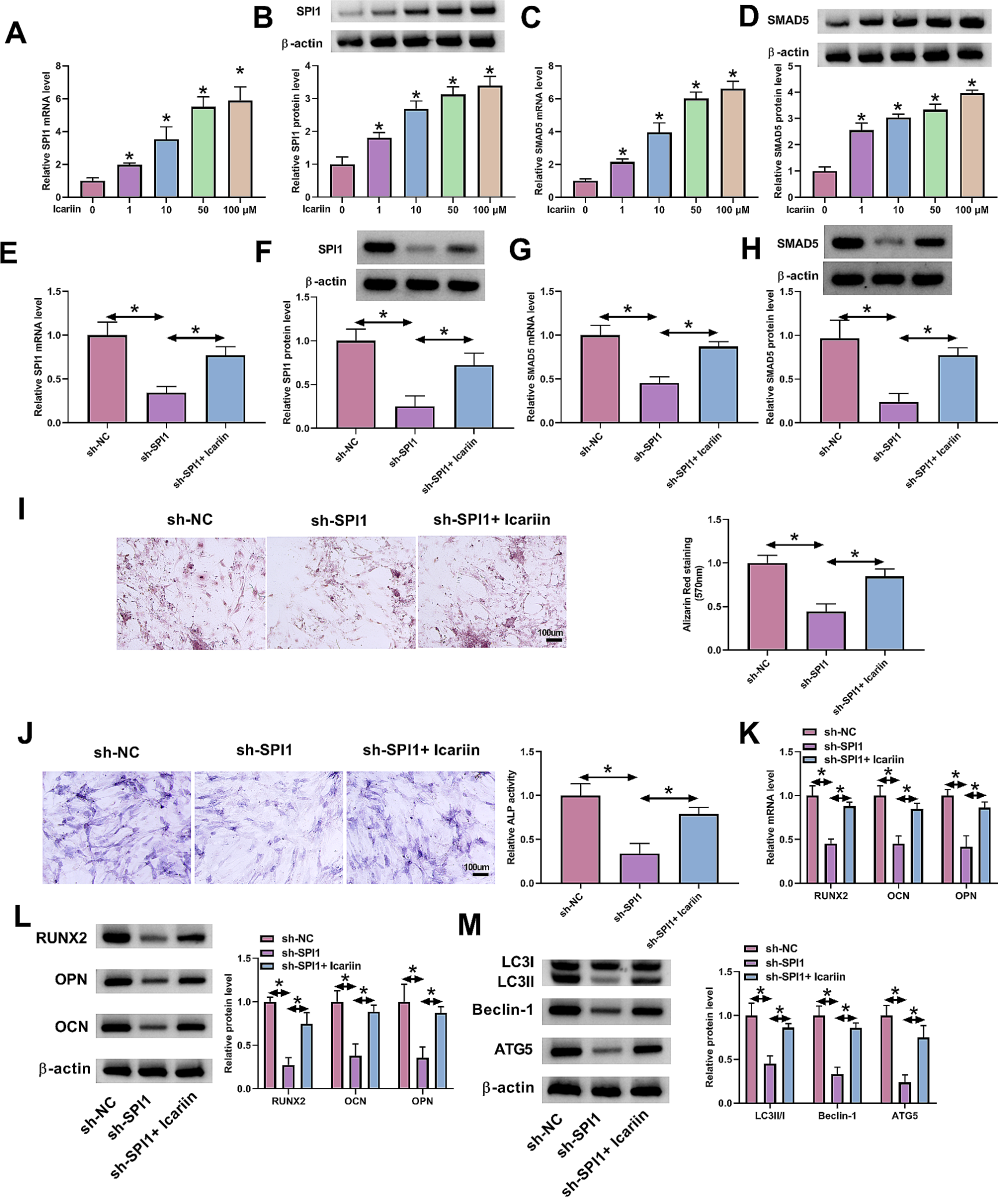
Icariin attenuates SPI1 depletion-mediated inhibition of osteogenic differentiation of MC3T3-E1 cells. **(A-D)** The evaluation of SPI1 (A and B) and SMAD5 **(C and D)** amount in MC3T3-E1 cells treated by different concentrations (0, 1, 10, 50, and 100 µM) of icariin. **(E-H)** The amount quantification of SPI1 **(E and F)** and SMAD5 **(G and H)** in MC3T3-E1 cells subjected to sh-NC transfection, sh-SPI1 transfection, or sh-SPI1 transfection + icariin treatment (50 µM, 48 h) accompanied by DEX stimulation. **(I-M)** The impacts of SPI1 loss-of-function or icariin treatment accompanied by SPI1 loss-of-function in the presence of DEX stimulation on in vitro calcium deposit (Alizarin Red staining assay) **(I)**, ALP activity (ALP activity assay) **(J)**, the amount of osteogenic markers (OPN, OCN and RUNX2) (K and L), and the changes of LC3II/LC3I ratio and Beclin-1 and ATG5 levels (immunoblotting) **(M)**. *n* = 3 independent biological replicates in A-M. *P < 0.05

Icariin, a natural product, has been well-known proven to possess the promoting ability in osteogenic differentiation [25, 37, 38] and thus represents a promising anti-osteoporosis drug [27, 29]. Our above data have demonstrated the promoting role of the SPI1/SMAD5 cascade in osteogenic differentiation of MC3T3-E1 cells. In an attempt to elucidate the molecular basis behind the function of icariin, we sought to examine whether the SPI1/SMAD5 cascade is responsible for icariin’s promoting ability in osteogenic differentiation. As expected, in MC3T3-E1 cells, icariin led to a remarkable augmentation in SPI1 and SMAD5 expression, as demonstrated by qPCR and immunoblotting (Fig. 6A and D). Moreover, in the presence of DEX stimulation, transfection of a shRNA targeting SPI1 (sh-SPI1) downregulated SPI1 and SMAD5 protein levels in MC3T3-E1 cells; however, icariin markedly rescued SPI1 and SMAD5 expression in the same conditions (Fig. 6E and H), suggesting the important regulation of icariin in the SPI1/SMAD5 cascade. Under DEX conditions, SPI1 depletion by sh-SPI1 diminished the calcium deposit and ALP activity of MC3T3-E1 cells, which could be rescued by icariin treatment (Fig. 6I and J). Similarly, SPI1 depletion resulted in a decreased expression of osteogenic markers OPN, OCN, and RUNX2 in MC3T3-E1 cells under DEX conditions, while icariin could attenuate these suppressions (Fig. 6K and L). In addition, depletion of SPI1 was validated to impede the autophagy of MC3T3-E1 cells under DEX conditions, as shown by the reduced ratio of LC3II/LC3I and downregulated protein levels of Beclin-1 and ATG5 following SPI1 depletion (Fig. 6M). Furthermore, icariin could reverse these changes induced by SPI1 depletion in MC3T3-E1 cells under DEX conditions (Fig. 6M). Taken together, our results suggest that icariin facilitates osteogenic differentiation of MC3T3-E1 cells, at least in part, through the SPI1/SMAD5 cascade.

## Discussion

Enhancement of osteogenic differentiation can contribute to osteoporosis remission [39, 40]. Developing pharmacological agents that drive osteogenic differentiation in osteoporosis remains challenging. The bioactive protective phytochemical icariin has become an anti-osteoporosis candidate in the research of pharmacological agents [27, 28]. Less insight, however, has been gained into the molecular basis underlying the protective role of icariin in osteoporosis. Here, we demonstrate that the SPI1/SMAD5 cascade is capable of inducing osteogenic differentiation of MC3T3-E1 cells, the murine pre-osteoblast cells that have been widely applied for the research of osteogenic differentiation in osteoporosis [41, 42]. Importantly, our findings suggest that the SPI1/SMAD5 cascade underlies the promoting effect of icariin on osteogenic differentiation of MC3T3-E1 cells. These findings in this report provide novel evidence for the pharmacological application of icariin in treating osteoporosis.

Through bioinformatics analysis, the transcription factor SPI1 is indicated as a possible diagnostic marker in osteoporosis [10]. Dysregulation of SPI1 is linked to the development of osteoporosis [43]. Recent work has revealed the important activity of SPI1 in facilitating osteogenic differentiation of bone marrow mesenchymal stem cells in acute suppurative osteomyelitis [11] and fibroblasts in ankylosing spondylitis [12]. However, whether SPI1 is involved in osteogenic differentiation of MC3T3-E1 cells is unknown. Our data show the upregulation of SPI1 during osteogenic differentiation of MC3T3-E1 cells. Indeed, we demonstrate the induction of increased SPI1 expression in osteogenic differentiation of MC3T3-E1 cells. Enhancement of autophagy contributes to osteogenic differentiation of MC3T3-E1 cells [32, 33]. Via analysis of autophagy-associated proteins, we reinforce the notion that SPI1 upregulation is able to induce osteogenic differentiation of MC3T3-E1 cells by activating autophagy. On the other hand, Liu et al. point to the ability of SPI1 to enhance osteoclast formation via the suppression of hepcidin [44]. The promoting activity of SPI1 in osteogenic differentiation and osteoclast formation suggests its intricate and multiple roles in bone regeneration.

One important question is how SPI1 induces osteogenic differentiation in MC3T3-E1 cells. In this report, we have disclosed that SPI1 enhances SMAD5 transcription and expression. Physiologically, SMAD5 possesses crucial functions in maintaining the balance of osteogenesis [45] and modulating osteogenic differentiation [14, 15]. It is well known that deregulation of SMAD5 participates in the pathogenesis of osteoporosis [17, 18], and increasing SMAD5 expression can promote bone formation during osteoporosis [20, 46]. Earlier documents have highlighted the promoting impact of SMAD5 on osteogenic differentiation of MC3T3-E1 cells [35, 47], which is also supported by our data that downregulation of SMAD5 diminishes osteogenic differentiation of MC3T3-E1 cells. Furthermore, we have confirmed the promoting function of SPI1 in osteogenic differentiation of MC3T3-E1 cells by upregulating SMAD5 for the first time. Thus, upregulation of SPI1 and SMAD5 expression may induce bone formation during osteoporosis.

Based on the induction of osteogenic differentiation during osteoporosis, the bioactive phytochemical icariin has become a promising anti-osteoporosis candidate [27, 28]. In MC3T3-E1 cells, icariin has been reported to contribute to osteogenic differentiation by modulating the Notch signaling and the circular RNA circ_0000349/miR-138-5p/JMJD3 cascade [37, 48]. An earlier study has unveiled the increased effect of icariin on SMAD5 expression in MC3T3-E1 cells [49]. In this report, we broaden the finding by demonstrating the enhancement of icariin in SMAD5 expression through the upregulation of SPI1. More importantly, we provide further evidence supporting the promoting function of icariin in osteogenic differentiation, depending on its modulation in the SPI1/SMAD5 cascade. Recent work has demonstrated that icariin can suppress osteoclast differentiation and diminish the levels of osteoclastogenesis-related factors, such as c-Fos, Trap, Ctsk, and NFATc1, in RANKL-induced RAW264.7 cells [50]. A previous study also shows that icariin can suppress the M2 phenotype polarization of Raw264.7-derived TAMs and attenuate their CCL5 expression by inhibiting SPI1, thereby suppressing osteoclast differentiation in the presence of the conditioned medium of tumor-associated macrophages and diminishing prostate cancer metastasis-induced bone destruction [51]. Our data show that icariin facilitates osteogenic differentiation of MC3T3-E1 cells by upregulating SPI1. With these findings, we hypothesize that the different regulatory patterns of icariin on SPI1 expression may be related to different cell types; that is to say, icariin may facilitate osteogenic differentiation by upregulating SPI1, whereas icariin may suppress osteoclast differentiation by inhibiting SPI1. More research about the effect of icariin on osteoclast differentiation will be warranted in further work. Furthermore, it is significant to study how icariin can affect both osteoblasts and osteoclasts together through a co-culture system, which is expected to be performed in further work. Additionally, in this study, our research findings by only using one mouse cell line are inadequate. To further demonstrate the mechanism, further investigations should be performed using primary osteoblast cells.

In summary, we have demonstrated that the SPI1/SMAD5 cascade, with the ability to enhance osteogenic differentiation, underlies the promoting effect of icariin on osteogenic differentiation of MC3T3-E1 cells. Our study provides novel evidence that icariin is a promising pharmacological agent to control osteoporosis progression.

### Electronic supplementary material

Below is the link to the electronic supplementary material.

Supplementary Material 1

Supplementary Material 2

## Data Availability

The analyzed data sets generated during the present study are available from the corresponding author on reasonable request.
