# Supplementary figures and images for: The SPI1/SMAD5 cascade in the promoting effect of icariin on osteogenic differentiation of MC3T3-E1 cells: a mechanism study
Source: J Orthop Surg Res. 2024 Jul 29;19:444. doi: 10.1186/s13018-024-04933-3 (PMC11285181; doi:10.1186/s13018-024-04933-3)

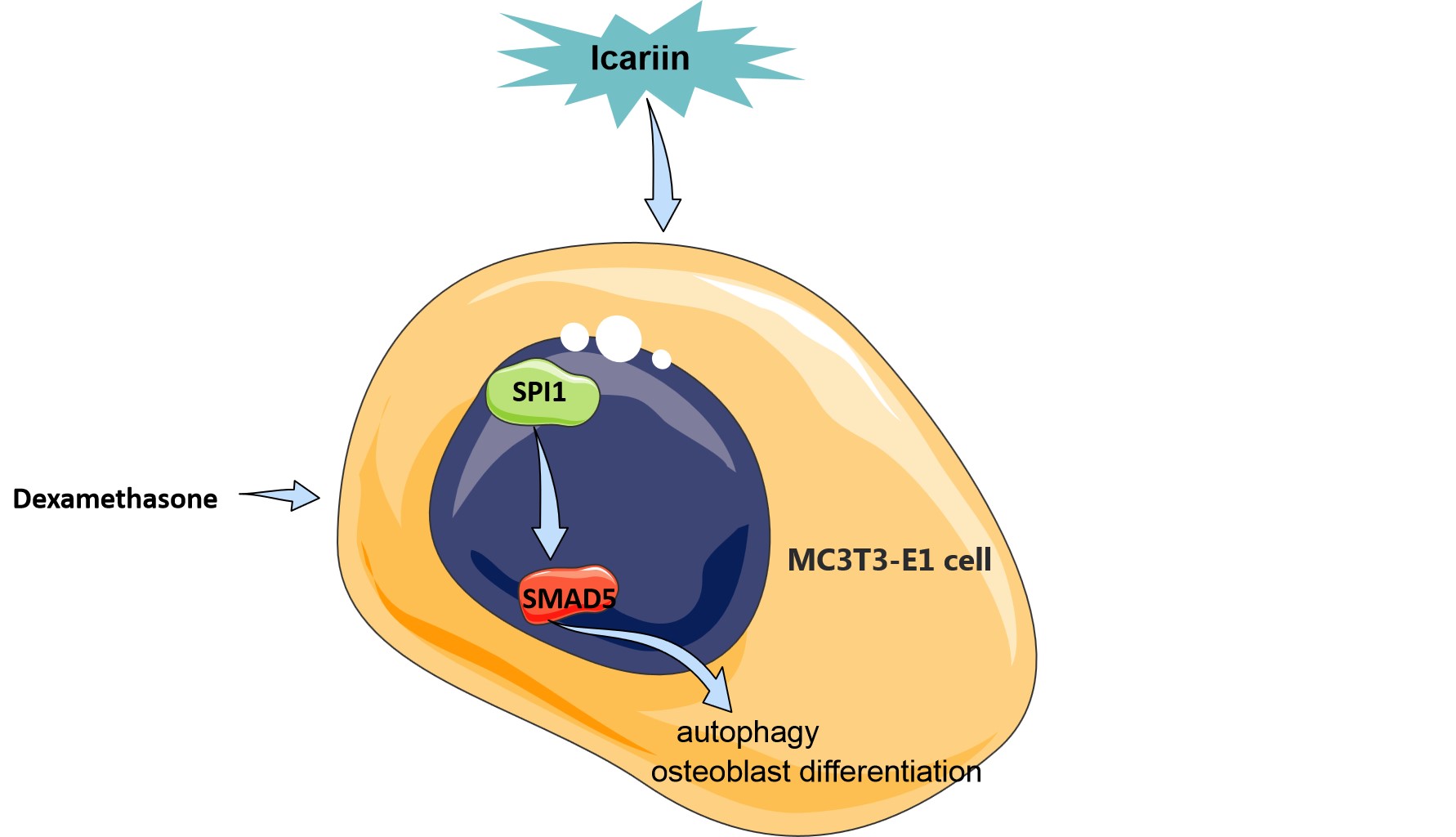

Supplement: Supplementary file 1 — Supplementary Material 1 [file 13018_2024_4933_MOESM1_ESM.jpg]
