# Supplementary material for: The SPI1/SMAD5 cascade in the promoting effect of icariin on osteogenic differentiation of MC3T3-E1 cells: a mechanism study
Source: J Orthop Surg Res. 2024 Jul 29;19:444. doi: 10.1186/s13018-024-04933-3 (PMC11285181; doi:10.1186/s13018-024-04933-3)
Supplement: Supplementary file 2 — Supplementary Material 2 [file 13018_2024_4933_MOESM2_ESM.docx]

**Graphical Abstract**

In dexamethasone-stimulated murine pre-osteoblast MC3T3-E1 cells, icariin upregulates SPI1 and thus enhances SMAD5 transcription, leading to enhanced autophagy and osteogenic differentiation of MC3T3-E1 cells.
